# Does providing the correct diagnosis as feedback after self-explanation improve medical students diagnostic performance?

**DOI:** 10.1186/s12909-019-1638-3

**Published:** 2019-06-11

**Authors:** M. Chamberland, J. Setrakian, C. St-Onge, L. Bergeron, S. Mamede, H. G. Schmidt

**Affiliations:** 1 Faculté de Médecine et des Sciences de la Santé, Université de Sherbrooke, 3001, 12e Avenue Nord, Sherbrooke, Qc J1H 5N4 Canada; 2 Institute of Medical Education Research Rotterdam, Erasmus Medical Center, Erasmus University, Rotterdam, The Netherlands; 3 Department of Psychology, Erasmus University, Rotterdam, The Netherlands

**Keywords:** Clinical reasoning, Feedback, Learning methods, Medical students, Self-explanation, Teaching methods

## Abstract

**Background:**

Self-explanation without feedback has been shown to improve medical students’ diagnostic reasoning. While feedback is generally seen as beneficial for learning, available evidence of the value of its combination with self-explanation is conflicting. This study investigated the effect on medical students’ diagnostic performance of adding immediate or delayed content-feedback to self-explanation while solving cases.

**Methods:**

Ninety-four 3rd-year students from a Canadian medical school were randomly assigned to three experimental conditions (immediate-feedback, delayed-feedback, control). In the learning phase, all students solved four clinical cases by giving i) the most likely diagnosis, ii) two main arguments supporting this diagnosis, and iii) two plausible alternative diagnoses, while using self-explanation. The immediate-feedback group was given the correct diagnosis after each case; delayed-feedback group received the correct diagnoses only after the four cases; control group received no feedback. One week later, all students solved four near-transfer (i.e., same final diagnosis as the learning cases but different scenarios) and four far-transfer cases (i.e., different final diagnosis from the learning cases and different scenarios) by answering the same three questions. Students’ diagnostic accuracy (score for the response to the first question only) and diagnostic performance (combined score of responses to the three questions) scores were assessed in each phase. Four one-way ANOVAs were performed on each of the two scores for near and far-transfer cases.

**Results:**

There was a significant effect of experimental condition on diagnostic accuracy on near-transfer cases (*p* < .05). The immediate-feedback and delayed-feedback groups performed equally well, both better than control (respectively, mean = 90.73, standard deviation =10.69; mean = 89.92, standard deviation = 13.85; mean = 82.03, standard deviation = 17.66). The experimental conditions did not significantly differ on far-transfer cases.

**Conclusions:**

Providing feedback to students in the form of the correct diagnosis after using self-explanation with clinical cases is potentially beneficial to improve their diagnostic accuracy but this effect is limited to similar cases. Further studies should explore how more elaborated feedback combined with self-explanation may impact students’ diagnostic performance on different cases.

**Electronic supplementary material:**

The online version of this article (10.1186/s12909-019-1638-3) contains supplementary material, which is available to authorized users.

## Background

Clinical reasoning is a complex skill that relies on an organized and interconnected knowledge base and requires time and repeated practice for students to acquire [1–4]. Designing effective teaching and learning interventions to support students in this endeavour remains a challenge for medical educators.

Despite the central importance of this educational issue, research data on specific approaches to teaching clinical reasoning in undergraduate programs are still surprisingly sparse [5]. Available evidence suggests that approaches oriented to teach the *process* of clinical reasoning (e.g., the hypothetico-deductive method) are not useful whereas interventions directed to help students build specific knowledge positively impact their diagnostic reasoning [5]. Self-explanation (SE) while solving clinical cases is among these knowledge-oriented instructional strategies [6–10].

Self-explanation is a learning strategy that requires students to engage actively with the learning material, providing for themselves specific explanations about its components, how these relate to each other and to their own prior knowledge, to deepen their understanding of the content [6]. Self-explanation promotes knowledge elaboration and monitoring and thus contributes to revision of knowledge representations in memory [6]. Recent research has investigated the effect of using self-explanation while solving clinical cases in medical students at the clerkship level [11]. In comparison to students not using SE while solving cases, diagnostic performance of students who self-explained improved one week later on new (transfer) clinical cases. This happened without any specific feedback either on the content or on the quality of their self-explanations. In that study, the improvement in diagnostic performance was restricted to topics with which students were less familiar, suggesting that SE is only useful when specific knowledge building is still in progress. Cases in topics that were less familiar to students seemed to offer them opportunities to revisit biomedical knowledge, increase coherence of their mental representations and facilitate transfer when facing new cases within the same topic [12]. The positive effect of SE on diagnostic reasoning seems to be further increased when students using SE with clinical cases are then exposed to self-explanation models generated by a junior resident on the same cases [13].

It seems therefore that exposing students to some additional content after SE may be beneficial for learning, depending on which information is provided to them. It remains unclear whether adding materials such as models to SE is worthwhile, considering the added cost of preparing modeling examples. The major strengths of using and implementing SE in its original form [11] include simplicity and low cost. The learning material consists basically of clinical cases, and once these have been developed, the SE activity can be used to exert its positive impact on students’ learning without any direct teacher supervision. The question is then, What type of additional content or feedback can be added to SE that would be low-cost, as well as providing additional benefits for learning?

Feedback as a whole is powerful but its effect varies considerably [14]. Feedback about the task, the outcomes, or knowledge of the results (corrective feedback) is useful in particular when it highlights students’ errors or misinterpretations but its content specificity may preclude its generalisation to other tasks. Feedback about the process allows deeper understanding, which may better help students with transfer tasks [14].

The timing of the feedback may also modulate its effectiveness [14]. Immediate feedback is provided immediately after a learner’s response whereas delayed feedback is given after minutes, hours or days [15, 16]. Although the issue of timing of feedback is still debated, there is evidence to suggest that delayed feedback may be more effective than immediate feedback, particularly for difficult items [16]. This benefit could be explained by the delay allowing the students to be exposed to the stimulus again, thus forcing them to process it a second time [14, 16].

While feedback is generally assumed to be beneficial, available evidence about the effect of combining SE and feedback on learning is in fact conflicting. In the context of computerised learning using worked-out examples (learning material that contains problem formulation, solution steps and the solution) or solved examples (learning material that contains only problem formulation and solution) it was shown that when instructional explanations were added to SE, students’ learning decreased as compared to SE alone [17]. An explanation for this was that because additional information on the problem was readily available, students’ SE, a strategy that supports generative activity, was turned off prematurely, leading to decreased learning outcomes [17]. In medicine, Heitzmann et al. [18] investigated the effect of combining SE prompts and adaptable feedback on diagnostic competence. In that experimental study, participants used worked-out examples of problems solved by novices in which errors were incorporated. SE prompts focused specifically on errors and the main outcome related to diagnostic competence was assessed by measuring the immediate effect on declarative-conceptual and decision-oriented practical knowledge. Adaptable feedback seemed beneficial for the latter type of knowledge whereas the addition of SE prompts did not lead to any significant further improvement.

In the perspective of optimizing the effect of SE as an instructional strategy while keeping this learning activity simple and practical for future implementation, we proposed to examine the effects on learning of feedback on the correct diagnosis and of its timing. The purpose of the present study was to assess the effect on medical students’ diagnostic performance of adding immediate or delayed content feedback, that is the correct diagnosis, to self-explanation while solving cases.

## Method

### Design

An experimental study consisting of a learning phase and an assessment phase was conducted with third-year medical students. In the learning phase, students solved clinical cases using self-explanation and received immediate feedback, delayed feedback or no feedback depending on the experimental condition to which they had been assigned. One-week later, in the assessment phase, all students solved in silence near and far transfer cases.

### Participants

Participants were third-year medical students from a Canadian university undergraduate program at clerkship level. This is a four-year curriculum with 2 ½ years of problem-based learning followed by 18 months of clerkship. Students from two consecutive cohorts were invited to take part in the study during the summer of 2015 and 2016. This choice aimed at reaching a sample size similar to previous studies from our group, in which the sample showed to be sufficient to identify effects of a similar learning strategy. [10–12]. During the summer, medical students on clerkship in the departments of Medicine and Paediatrics at our institution attended a mandatory summer learning activity consisting of solving clinical cases using self-explanation. Jiewere invited to participate in the study on a voluntary basis. Written consent was obtained from each student in order to use the data collected during this learning activity to answer the research question. This project was approved by the Ethics and Research - Education, and Social Sciences Committee at our institution (# CÉR-ESS-2015-14).

### Material

#### Clinical cases

In total, sixteen written clinical cases were used in the study; twelve jaundice cases and four cases about different topics. In the learning phase, only four cases were used, all on jaundice. In the assessment phase, twelve cases were used: four near-transfer jaundice cases (same final diagnosis as the learning cases but different scenarios), and four far-transfer jaundice cases (i.e., different final diagnosis from the learning cases and different scenarios) and the four cases on different topics (syncope, fever, leg swelling, renal failure). The latter cases were intercalated with the jaundice cases to reduce the possibility for students to recognise easily the diseases presented in the learning phase. All cases were developed by clinician teachers who have a long experience with the subjects and the population studied. These cases have been used in previous studies, and enabled to detect differences in students’ performance [13]. The iterative review of the cases by these experts, and their previous use suggest that they are targeted at the right level. Additional file 1 presents an example of a clinical case. The specific diagnoses of all the cases used in both phases of the study are presented in the Additional file 2.

#### Feedback

The feedback provided to the students in the first two groups (immediate feedback and delayed feedback) was simple content feedback consisting only of the correct final diagnosis for each of the four learning cases followed by a prompt to ensure students would process the received information. The prompt was the following: *Based on this information, and regardless of your previous answer, please take some time now to review your thought processes during self-explanation.* Immediate feedback (each correct diagnosis) was given after each case and delayed feedback (all four correct diagnoses) was given after the students had finished solving the four cases.

#### Sociodemographic survey

At the end of the assessment phase, a short questionnaire was used to collect sociodemographic data about the students and some information about their exposure to the topic of jaundice during the intervening week of the study.

### Procedure

We received a list of all students registered in the Medicine and Pediatric clerckships during the Summer 2015 and 2016. A research assistant used Excel to first assign a random number to students, than sort the names according to these numbers, and finally assign students to groups 1, 2 and 3 consecutively. Three groups were therefore formed: Group: 1- Immediate Feedback; Group 2- Delayed Feedback; and Group 3- Control. Three research assistants (RA) conducted the sessions with the participants for both phases of the study. To ensure a rigorous and standardised process, each RA followed a detailed written procedure on how to run each of the sessions in both phases. One RA had already conducted similar studies in the past trained the other RAs, and they practiced with each other before meeting with a first participant.

#### Learning phase

In the learning phase, a RA met each student individually. Students in all groups were first introduced to self-explanation as the learning strategy to use during this phase. More specifically, students were given a definition of SE, and they then listened to an audio example of SE based on a clinical vignette of a similar format to that used in the study but on a different topic. They were also made familiar with the three following questions to answer after each of the cases. 1-What is the most likely diagnosis? 2- What are the two main arguments supporting this diagnosis? 3- List two plausible alternative diagnoses.

The procedure differed according to the group students were allocated to. In the Immediate Feedback Group, students did self-explanation on a case for eight minutes. More specifically, they had to read out loud the case and generate freely explanations to themselves about the different elements included in the case, e.g. by returning to basic mechanisms, making links between clinical elements, generating general or specific diagnoses etc. During this phase, no feedback neither on self-explanation nor on content was provided to students. Then they had two minutes to write their answers in silence to the three questions. Immediately after, the research assistant gave to students the feedback (correct diagnosis) in a written form with the prompt. Students had 1.5 min to verbalize in response to that prompt while having access to the clinical case. Then, they self-explained about the second case, answered the questions and received the second feedback, and so on for the two other cases.

Students in the Delayed Feedback Group self-explained about the first case (8-min) then answered the questions (2-min) exactly as the previous group did but went on for the four cases before receiving any content feedback. When they had completed the four cases, the correct diagnoses of the four cases with the prompt were provided. They then had 1.5 min to go back to each case and verbalize in response to the prompt.

In the Control Group, students self-explained (8 min) and answered the three questions (two minutes) for each of the four cases in a similar way to the other two groups. However, they did not receive any feedback on the correct diagnoses. To equalize the time on task, the students were requested to solve a word puzzle for 1.5 min after each case. The learning phase lasted 1 h15 minutes for all the groups.

#### Assessment phase

For the assessment phase, students were met one week after the learning phase. They were asked to solve in silence twelve cases, eight jaundice cases (four near-transfer and four far-transfer cases) and four intercalated cases on other topics, by answering the same three questions asked during the learning phase. They had up to two hours to complete the twelve cases, and there was no time limit to spend on each case. They were asked to fill out the sociodemographic survey at the end of the session.

For both the learning and assessment phases, the cases were presented in a booklet with one case per page with the three questions on the opposite page. There were two versions of the booklet given alternately to the students in each group, one with cases in a particular order and the other with the cases in the reverse order. At the end of the study, the correct diagnoses of the learning cases were also provided to students in the control group.

### Analyses

One investigator (MC) who was blind to the students’ condition corrected all students’ responses with a correction grid that has been used in previous studies [11, 13]. In these studies by our research group, clinician teachers developed the three questions that students answered for each case, which were considered to give an indication of what the diagnostic process in real clinical practice entails. Each question was corrected, in these previous studies, by using a 3-point scale, ranging from 0 to 2 (0 if the answer was incorrect; 1 if partly correct; and 2 if entirely correct). The reliability of the scoring procedure proved to be acceptable, with inter-rater reliability measured by intra-class correlations ranging from 0.70 to 1.00 in the previous studies. The same correction grid was adopted to assess the students’ responses in the present study.

As was done in our previous studies, we calculated two different scores to assess students’ performance. The diagnostic accuracy score, which assesses the accuracy of the students’ final diagnosis, was the score obtained for the first question (1-What is the most likely diagnosis?; for a maximum of 2 points per case). The diagnostic performance score was the sum of the scores obtained for the three questions (maximum of 6 points per case). That score is intended to provide a more global measure of the quality of students’ clinical reasoning. We then summed diagnostic accuracy scores and diagnostic performance scores for the four learning cases, the four near-transfer cases and the far-transfer cases. Theses sums were then converted to percentages.

Descriptive statistics (frequencies, means and standard deviations) were used to report the different results below. We conducted one-way ANOVAs to assess differences between groups for age and level of confidence of students’ approach to jaundice, and we conducted a Pearson chi-square test to look at differences between groups for gender, clerkship rotation, number of hours of personal work on jaundice, and for the number of hours of teaching related to jaundice.

We conducted two one-way ANOVAs on the scores at the learning phase, one on diagnostic accuracy and one on diagnostic performance, to ensure that our groups were comparable on their performance at the beginning of the study. We conducted four separate one-way ANOVAs (on diagnostic accuracy score on near-transfer cases, diagnostic accuracy score on far-transfer cases, diagnostic performance score on near-transfer cases, and diagnostic performance score on far-transfer cases) to assess the impact of adding feedback to self-explanation on performance. With ANOVAs, we also wanted to assess the impact of the timing of the feedback (immediate vs delayed) on the students’ performance. In the event of significant ANOVA results, we planned to use Tukey post hoc tests to assess where differences between groups lay.

## Results

Ninety-four students participated in the study (*n* = 54/59 in 2015) and (*n* = 40/40 in 2016). The number of participants in each group was the following: Immediate Feedback = 31, Delayed Feedback = 31, and Control group = 32. Fifty-seven percent of the sample were women (*n* = 53). The mean age of students was 23.27 years (3.10).

Table 1 presents sociodemographic data for each group (age, gender, clerkship rotation and relevant information about their exposure to the topic of jaundice during the intervening week of the study). There were no differences between groups in age (*F*[2, 89] = 0.48, *p* = .622), gender (χ^2^ [2] = 0.405, *p* = .817), clerkship rotation (χ^2^ [4] = 0.41, *p* = .354), personal study on jaundice (χ^2^ [4] = 1.60, *p* = .809), teaching received related to jaundice (χ^2^ [4] = 1.11, *p* = .893), nor on the students’ level of confidence about their approach to jaundice (*F*[2, 85] = 0.960, *p* = .387).

**Table 1:** Sociodemographic data as a function of groups

		Immediate Feedback Group	Delayed Feedback Group	Control Group
Mean age (SD)		23.58 (3.97)	23.40 (3.10)	22.84 (1.93)
Gender		W = 53.3%	W = 61.3%	W = 56.3%
		M = 46.7%	M = 38.7%	M = 43.7%
Clerkship rotation	Internal medicine	19.4%	6.5%	12.5%
	Specialty of internal medicine	38.7%	29.0%	40.6%
	Pediatrics	41.9%	64.5%	46.9%
Personal study on jaundice during the week	0 h	64.5%	64.5%	54.8%
	≤ 2 h	19.4%	22.6%	32.3%
	> 2 h	16.1%	12.9%	12.9%
Teaching related to jaundice during the week	0 h	67.7%	67.7%	73.3%
	≤ 2 h	25.8%	22.6%	23.3%
	> 2 h	6.5%	9.7%	3.3%
Mean level of confidence regarding approach to jaundice (SD)		5.86 (1.43)	6.34 (1.52)	6.02 (1.09)

### Learning phase performance

Table 2 presents means and standard deviations for both scores of the learning phase. The one-way ANOVAs showed that there were no differences between groups for the diagnostic accuracy score (*F*[2, 91] = 0.25, *p* = .782), nor for the diagnostic performance score (*F*[2, 91] = 0.47, *p* = .624).

**Table 2:** Mean diagnostic accuracy and diagnostic performance scores for the learning cases

Scores	Immediate Feedback Group Mean (SD)	Delayed Feedback Group Mean (SD)	Control Group Mean (SD)	*p-*value of the ANOVA
Diagnostic accuracy Scores	72.58 (21.51)	68.95 (18.50)	71.09 (21.17)	.782
Diagnostic performance Scores	56.18 (16.13)	56.45 (12.16)	59.38 (14.82)	.624

### Assessment phase performance

#### Diagnostic accuracy scores on near and far-transfer cases

Table 3 presents the means and standard deviations for the diagnostic accuracy score on near and far-transfer cases as a function of experimental condition. The one-way ANOVAS showed a significant difference between groups for the near-transfer cases (*F*(2, 91) = 3.53, *p* = .033, *r* = 0.27), but no difference between groups for the far-transfer cases (*F*(2, 91) = 0.46, *p* = .630). The Tukey post hoc test revealed differences between the Control Group and Immediate Feedback Group (*p* = .048), but not between the Control Group and Delayed Feedback Group (*p* = .081), nor between the Immediate Feedback Group and the Delayed Feedback Group (*p* = .974).

**Table 3:** Mean diagnostic accuracy scores on near- and far- transfer cases

Scores	Immediate Feedback Group Mean (SD)	Delayed Feedback Group Mean (SD)	Control Group Mean (SD)	*p-*value of the ANOVA
Scores on near-transfer cases	90.73 (10.69)	89.92 (13.85)	82.03 (17.66)	.033
Scores on far-transfer cases	49.60 (23.60)	48.79 (23.79)	53.91 (20.68)	.630

#### Diagnostic performance scores on near and far-transfer cases

Table 4 presents the means and standard deviations for the diagnostic performance scores on near and far-transfer cases. The one-way ANOVAS showed no significant difference between groups for the near-transfer cases (*F*(2, 91) = 0.21, *p* = .810), nor the far-transfer cases (*F*(2, 91) = 0.53, *p* = .591).

**Table 4:** Mean diagnostic performance scores on near- and far-transfer cases

Scores	Immediate Feedback Group Mean (SD)	Delayed Feedback Group Mean (SD)	Control Group Mean (SD)	*p-*value of the ANOVA
Scores on near-transfer cases	69.22 (9.72)	67.47 (11.36)	69.01 (13.34)	.810
Scores on far-transfer cases	51.88 (15.55)	50.54 (17.04)	54.56 (14.83)	.591

## Discussion

In this experimental study, we studied the effect of adding immediate or delayed content feedback to self-explanation on diagnostic performance and diagnostic accuracy in medical students at clerkship level. The feedback was directed to the task and consisted of providing the correct diagnosis for each of the clinical cases solved by the students followed by a prompt inviting them to review consequently their reasoning.

The results show that one week later, in the assessment phase, the immediate feedback group obtained significantly higher diagnostic accuracy scores on near transfer cases than the control group. The different time in which feedback was provided did not affect diagnostic accuracy. In addition, there was no difference between the groups for diagnostic performance score on near transfer cases as well as no difference on either score on far transfer cases.

Although teachers assume feedback is always useful, evidence points to its effectiveness being dependent on a variety of factors such as the focus of feedback, and the cognitive processes it triggers in the student [14]. Corrective feedback alone represents a simple type of feedback directed to the task. Combined with a prompt in our study, it might have provided students with the opportunity to process the information by briefly revisiting their diagnosis. A correct answer being specific to the problem at hand, is content-related and may be more difficult to generalise or subsequently apply to new cases [14]. Given this, it is not surprising that the effect only appeared on diagnostic accuracy on near transfer cases and not on other outcome measures. Beyond the correct diagnosis, students may require more elaborate feedback, since they need to process complex data and to achieve deeper learning – beyond simple memorization – in order to solve far transfer cases and build arguments to justify their differential diagnoses [19].

Indeed, a closer look at the cases used in the study and what was required from the students may help explain the effect found for near transfer cases only. As defined and operationalized in our study, near transfer cases used in the assessment phase were similar to the learning cases, with the same key clinical data relevant to the final diagnosis and same final diagnosis but with modifications of “superficial or contextually irrelevant” data for the specific disease (e.g.: age, gender, past medical history, medications, occupation). In contrast, the far transfer cases consisted of problems that differed substantially from the ones seen in the learning phase. Although the far transfer and learning cases were related and share common general underlying pathophysiologic processes (e.g. cholestasis), they refer to different clinical scenarios linked to different specific diseases (e.g. pancreatic cancer vs common bile duct stones) [13, 20]. The diagnostic accuracy score comprises only the specific diagnosis whereas the diagnostic performance score combines the specific diagnosis, two arguments supporting this diagnosis and two plausible alternatives diagnoses. Therefore, providing a diagnosis for a similar case of the same disease may mainly involve simple recall or pattern recognition. Conversely, having to justify a diagnosis, even if the case is similar, or having to solve (and/or justify) a different case might force the student to engage in a deliberate process with systematic analysis of data, to make sense of the clinical information, and to assess how individual elements fit with one of a set of generated possible diagnoses [21]. When the feedback given at the end of the exercise is limited to providing the correct diagnosis, it may mainly help students to validate/reinforce or put the right label on a constellation of symptoms and signs and may not necessarily help them to process the clinical information at a deeper level later when facing similar or different cases. This effect of adding corrective feedback may be construed as limited because it translates only to a better diagnostic accuracy score for near transfer cases. However, an argument can be made that if this basic type of content feedback combined with prompt helps students to diagnose correctly similar cases in future, this limited effect is still probably educationally – and ultimately clinically – important. In fact, diagnosing patients by recognising similarities with previous cases is critical for clinicians in practice [4].

In a previous study on students’ SE using a similar experimental design, we investigated the added value of listening to residents’ SE on medical students’ diagnostic performance [13]. In that study, medical students solved clinical cases with SE and then had to listen (or not) to a resident’s SE about the same case, with or without prompts. The self-explanations expressed out loud by the residents while solving the problem represented how they approached the cases, how they progressively interpreted or tried to integrate the clinical information, but the student did not have access to the end of the residents’ self explanations, (i.e., the answers: final diagnosis, main arguments or top two alternative diagnoses). In that study, we presented the residents’ SE within the theoretical framework of example-based learning [22]. However, it might also be interesting and relevant to the present discussion to alternatively view residents’ SE as an external source of feedback. This feedback could then be described as mainly focussing on the process of diagnosing the specific case instead of simply providing the final correct answer. In contrast to the present study, that study showed a significant positive effect of listening to residents’ SE with prompts on diagnostic performance on both near and far-transfer cases. The residents’ self-explanation on how they reasoned through the case is possibly therefore a more powerful type of feedback than the one we used in the present study. While this is purely conjectural, research on feedback has indeed suggested that feedback directed to the process provides students with opportunities for deep data processing, thus enhancing transfer of learning [14].

Regarding the effect of feedback according to provision timing, we observed no significant difference between immediate and delayed feedback on students’ diagnostic reasoning in the present study. Only the immediate feedback group, however, outperformed the control group, which suggests that students may have benefitted more from it than from the delayed feedback. We cannot say if this is due to any detrimental influence of the process required to make use of delayed feedback or to operational issues related to our study. In order to process the delayed feedback, students first have to “clear” from memory the clinical case with which they are currently engaged, then actively retrieve the previous case to which the feedback refers and start again reflecting on that case by trying to integrate the external information provided in the feedback. If the potential benefit of the delay is linked to this cognitive extra work [14, 16] it would probably take more time to process delayed feedback compared to immediate feedback. In the present study, students in the delayed or immediate conditions had the same total time on task, which was strictly controlled. This might be an explanation for the absence of difference we observed.

This study has some limitations. It was conducted within strictly controlled conditions and, therefore, its results may not generalise to real clinical settings. However, educational approaches such as SE possibly have a higher potential during practice with simulated cases, taking place in an environment similar to that in our study. Additionally, since students’ knowledge evolves during their training, the findings we observed in this study with third year medical students at clerkship might have been different for learners at other levels of training. On a statistical point of view, when conducting the four different ANOVAs we did not use a Bonferonni correction on the *p* value. Applying this correction, the *p*-value would have been of .0125, leading to a non-significant effect of immediate feedback on diagnostic accuracy score on near-transfer cases.

## Conclusion

The results of this study suggest that providing the correct diagnosis, a simple content feedback, immediately after students’ use of self explanation on clinical cases improves their subsequent ability to correctly diagnose similar cases. These findings have practical implications and could inform the effective design of educational activities that use self-explanation to support the development of students’ diagnostic reasoning. Adding simple corrective feedback in the form of the correct diagnosis and making sure that students have the opportunity to process it seems a very simple measure to improve specifically students’ diagnostic ability for similar cases. Further studies are needed to explore the effect of alternative types of feedback directed to the task or the process and to assess how this may influence learning at a deeper level. Regarding the issue of timing, longer delays before giving feedback allowing sufficient time for the student to return to the problem deserve further study as well.

## Additional files

Additional file 1: Example of a clinical case. Description of data: the file shows an example of a clinical case used in the study. (DOCX 26 kb)

Additional file 2: Clinical cases used in the study. Description of data: the file shows a table with the diagnoses of the clinical cases used in the study. (DOCX 13 kb)

## Data Availability

The datasets used and/or analysed during the current study are available from the corresponding author on reasonable request.

## Abbreviations

RA: Research assistant
SD: Standard deviation
SE: Self-explanation
