# Supplementary material for: Does providing the correct diagnosis as feedback after self-explanation improve medical students diagnostic performance?
Source: BMC Med Educ. 2019 Jun 11;19:194. doi: 10.1186/s12909-019-1638-3 (PMC6558772; doi:10.1186/s12909-019-1638-3)
Supplement: Supplementary file 1 — Example of a clinical case. Description of data: the file shows an example of a clinical case used in the study. (DOCX 26 kb) [file 12909_2019_1638_MOESM1_ESM.docx]

**Additional file 1**

Example of a clinical case

*A 76-year-old man presents with progressive jaundice.*

***HPI:*** *The patient noted a gradual yellow-green discoloration of his skin over three weeks. He has no abdominal pain other than a vague epigastric discomfort which he has experienced after meals. He noticed that his urine have become dark, and his stools have become pale but he reports no diarrhea or constipation. His appetite seems to be diminished and he reports having lost 5-7 kg over the past month. He denies fever, chills, or night sweats. There is no travel history and no change in his dietary habits.*

***PMH:*** *Systolic hypertension and type 2 diabetes mellitus, both for 1 year. One episode of acute diverticulitis in 2006. Superficial thrombophlebitis of the right lower extremity 2 months ago.*

***Medications:*** *Hydrochlorothiazide 12, 5 mg QD, Metformin 250mg BID.*

***Habits:*** *A* ***r****etired carpenter, he still lives in his own house. He was widowed 3 years ago. There is no history of smoking or of alcohol use.*

***Physical examination:*** *The patient’s general appearance is normal except for the presence of a deeply jaundiced skin, BP 145/75, Pulse 75, afebrile, weight 75kg. There is no enlargement noted in any of the lymph node groups. Cardiac examination is within normal limits. Lung examination is normal. On abdominal examination, there is no tenderness noted. The liver and spleen are not enlarged. There are no palpable masses. The rectal examination is normal. There is no edema of the lower extremities.*

***Laboratory investigations :*** *CBC : hemoglobin 125 g/L(130-180) MCV 88 fL(80-100) WBC 6,2 X 10^9^/L(3,8-10,6) Platelets 350 X10^9^/L(130-400); Serum electrolytes and creatinine are normal; Fasting blood sugar is 6,9 mmol/L(3,3-6,1); AST 52 IU/L (14-50) , ALT 75 IU/L (21-72), alkaline phosphatase 625 IU/L(43-200), total bilirubin 110umol/L (3,4-17); direct bilirubin 100umol/L; albumin 33g/L (35-50); INR and PTT normal. Urinalysis: the urine is brown, testing strongly positive for bilirubin, and negative for urobilinogen.*
