# Supplementary material for: Does providing the correct diagnosis as feedback after self-explanation improve medical students diagnostic performance?
Source: BMC Med Educ. 2019 Jun 11;19:194. doi: 10.1186/s12909-019-1638-3 (PMC6558772; doi:10.1186/s12909-019-1638-3)
Supplement: Supplementary file 2 — Clinical cases used in the study. Description of data: the file shows a table with the diagnoses of the clinical cases used in the study. (DOCX 13 kb) [file 12909_2019_1638_MOESM2_ESM.docx]

**Additional file 2**

Clinical cases used in the study

| **Broad categories of diseases** | **Specific diagnosis** | | | |
| --- | --- | --- | --- | --- |
|  | **Learning phase** |  | **Assessment phase** | |
|  | **Training cases** |  | **Near-transfer cases** | **Far-transfer cases** |
| 1-Acute hepatitis | Viral Hepatitis B |  | Viral Hepatitis B | Viral Hepatitis A |
| 2-Cirrhosis | Chronic alcoholism |  | Chronic alcoholism | Hemochromatosis |
| 3-Obstructive jaundice | Pancreatic tumor |  | Pancreatic tumor | Choledocholithiasis |
| 4-Hemolysis | Auto-immune, idiopathic |  | Auto-immune, idiopathic | Cold agglutinins, Mycoplasma infection |
| Cases on other topics  1-Syncope  2-Fever  3-Leg swelling  4-Renal failure |  |  | Bleeding gastric ulcer  Acute prostatitis  Venous thrombosis  Acute tubular necrosis | |
